# Mindbomb 2 is dispensable for embryonic development and Notch signalling in zebrafish

**DOI:** 10.1242/bio.014225

**Published:** 2015-10-30

**Authors:** Shohei Mikami, Mizuki Nakaura, Atsuo Kawahara, Takamasa Mizoguchi, Motoyuki Itoh

**Affiliations:** 1 Graduate School of Pharmaceutical Sciences, Chiba University, Chiba 260-8675, Japan; 2 Laboratory for Developmental Biology, Center for Medical Education and Sciences, Graduate School of Medical Science, University of Yamanashi, Yamanashi 409-3898, Japan

**Keywords:** Mib2, Notch signalling, Neuronal development, Zebrafish, Ubiquitin ligase

## Abstract

The Mindbomb E3 ubiquitin protein ligase (Mib) family of proteins, Mib1 and Mib2, are RING finger ubiquitin ligases that share specific substrates. Mib1 is known to play essential roles in Notch signalling by ubiquitinating Notch ligands *in vivo*. Conversely, the functions of Mib2 *in vivo* are not fully understood, although Mib2 ubiquitinates multiple substrates, including Notch ligands, *in vitro*. To determine the Notch-dependent and Notch-independent functions of Mib2 *in vivo*, we generated mutant alleles of zebrafish *mib2* using transcription activator-like effector nucleases (TALENs). We found that *mib2* homozygous mutants were viable and fertile. Notch-mediated functions, such as early neurogenesis, somitogenesis, and pigment cell development, were not affected in *mib2* mutant embryos. The lack of Notch-deficient phenotypes in *mib2* mutants was not due to compensation by a *mib2* maternal gene product because *mib2* maternal-zygotic mutants also did not exhibit a distinct phenotype. We also showed that Mib2 does not redundantly act with Mib1 because the genetic ablation of *mib2* neither enhanced *mib^tfi91^*-null phenotypes nor did it alleviate antimorphic *mib^ta52b^* phenotypes. Furthermore, the postulated Notch-independent roles of Mib2 in maintaining muscular integrity and N-methyl-D-aspartate receptor (NMDAR) activity were not evident: *mib2* mutants did not show phenotypes different from that of the control embryos. These observations suggest that Mib2 is dispensable for embryonic development and does not have redundant functions with Mib1 in Notch signalling at least during early development stages in zebrafish.

## Abstract (precis)

**Summary:** Mib2 is dispensable for embryonic development and does not have redundant functions with Mib1 in Notch signalling, at least during early development stages in zebrafish.

## INTRODUCTION

Ubiquitination is a posttranslational modification that regulates protein functions and is associated with diverse cellular functions and human diseases (Komander and Rape, 2012; Popovic et al., 2014). E3 ubiquitin ligases play important roles in facilitating ubiquitin transfer from the ubiquitin-conjugating enzyme E2 to the substrate and determining substrate specificity (Pickart, 2001).

Mib2, which is also known as skeletrophin, is a RING finger ubiquitin ligase that was originally cloned from aggregated neuroblastoma cells and shown to interact with alpha-actin (Takeuchi et al., 2003). The domain organization and sequence of Mib2 are similar to those of Mib1, which plays essential roles in activating Notch signalling in metazoans (Itoh et al., 2003; Koo et al., 2005; Nguyen et al., 2007; Zhang et al., 2007a; Yamamoto et al., 2010). Notch signalling is a well-conserved signalling pathway that is required for several processes during embryonic development, such as the generation of neurons and somites, and adult tissue function (Guruharsha et al., 2012). Previous studies showed that Mib2 ubiquitinates Notch ligands *in vitro* (Koo et al., 2005; Takeuchi et al., 2005; Zhang et al., 2007a). However, Mib2 may not significantly affect Notch signalling *in vivo* because mouse or *Drosophila Mib2* mutants do not show strong Notch loss-of-function phenotypes. Because maternal gene products deposited into eggs are involved in early embryonic development in metazoan, the lack of a strong phenotype of *mib2* mutants may be due to the masking of phenotypes in homozygous *mib2* mutants by a Mib2 maternal gene product during development. However, the maternal/zygotic function of Mib2 has not been explored.

Moreover, because Mib2 is homologous to Mib1, redundant functions between Mib1 and Mib2 have been examined using double knockout/knockdown animals. In mice, Mib1/Mib2 double knockout does not cause a more severe phenotype than Mib1 single knockout. In contrast Zhang et al. showed the roles of Mib2 and Mib1 are redundant in zebrafish, as evidenced by the fact that *mib2* knockdown enhances the phenotypes of *mib1*-null mutant embryos. These conflicting reports may indicate species differences in Mib2 functions. Alternatively, the morpholino (MO)-mediated knockdown of Mib2 in zebrafish might cause non-specific effects, as evidenced by a recent report demonstrating that MO-mediated knockdown is associated with a high false-positive rate (Kok et al., 2015). Thus, zebrafish *mib2* mutant analysis should help resolve this issue.

RING-type E3 ubiquitin ligases regulate diverse cellular functions, and a RING ubiquitin ligase may have multiple substrates (Metzger et al., 2014). Indeed, previous studies suggested that Mib1/Mib2 family ubiquitin ligases have multiple substrates other than the Notch ligand; thus, Mib2 may also play Notch signalling-independent roles *in vivo* (Tseng et al., 2014). Accordingly, Mib2 is involved in maintaining muscle integrity *in vivo* in *Drosophila*, although the Mib2 substrate responsible for this function is not known (Nguyen et al., 2007; Carrasco-Rando and Ruiz-Gomez, 2008). In contrast, the conservation of this function of Mib2 among different species is not well understood. Conversely, the N-methyl-D-aspartate receptor (NMDAR) NR2B subunit was identified as a substrate for Mib2 *in vitro*, but the role of Mib2 in regulating NMDAR activity has not been investigated *in vivo* (Jurd et al., 2008).

Here, we generated zebrafish *mib2* mutant lines using Transcription activator-like effector nucleases (TALENs) to better understand the *in vivo* role of Mib2. An analysis of the *mib2* mutants did not reveal obvious contributions of Mib2 to Notch signalling, muscle integrity, and NMDAR activity. Furthermore, redundant roles between Mib1 and Mib2 in Notch signalling were not explicitly evident, at least during early development.

## RESULTS

### Generation of Mib2 mutant zebrafish

**Fig. 1.**
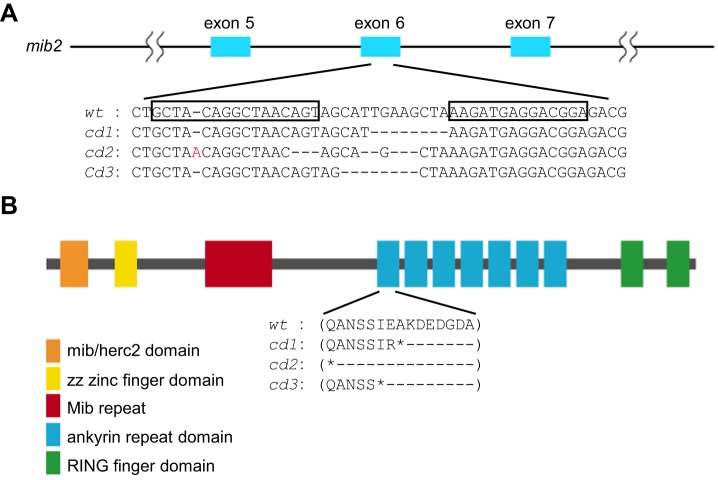
**Generation of *mib2* mutants by TALEN.** (A) Schematic representation of the genomic structure of the *mib2* gene and mutations produced by TALEN. The TALEN target sequences are boxed. (B) Domain organization of Mib2 protein. All three mutations (*cd1, cd2,* and *cd3*) generate premature stop codons.

**Fig. 2.**
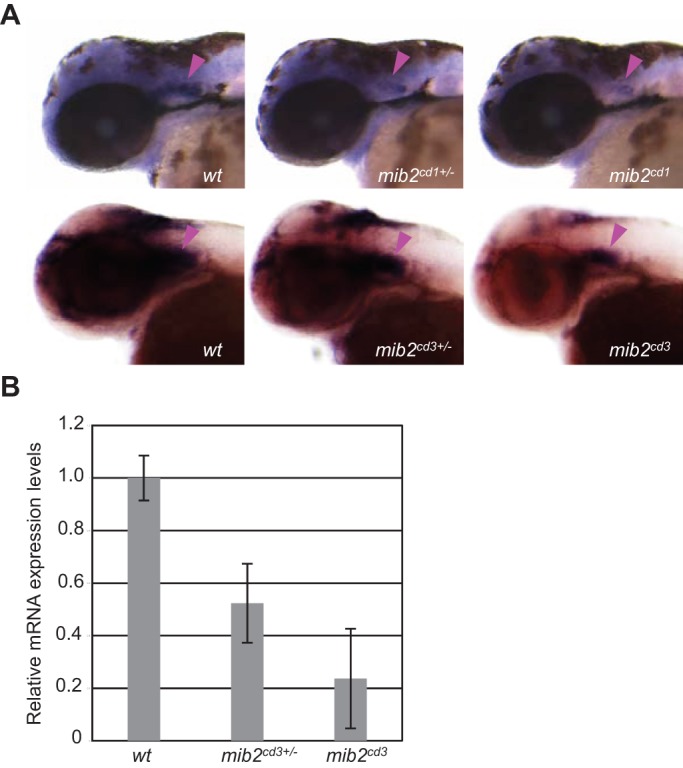
**Expression of *mib2* is reduced in *mib2* mutant embryos.** (A) Whole-mount *in situ* hybridization using the *mib2* antisense probe in embryos at 48 hpf. Arrowheads indicate *mib2* expression in the ear. Head region, side views of embryos at 48 hpf with anterior to the left. (B) Relative expression level of *mib2* mRNA measured by q-PCR in the 48 hpf-embryos. Error bars represent the mean±s.d. of three independent experiments.

To understand the functions of Mib2 *in vivo*, we generated three mutant alleles of zebrafish *mib2* using transcription activator-like effector nucleases (TALENs), i.e. *mib2^cd1^*, *mib2^cd2^* and *mib2^cd3^* (Fig. 1A,B). Two of the mutant alleles, *mib2^cd1^* and *mib2^cd3^*, feature 8 bp deletions, whereas *mib2^cd2^* features one bp insertion and 8 bp deletions (Fig. 1). All mutations created stop codons in the first ankyrin repeat domain (Fig. 1A,B) (Mib2^cd1^, A538stop; Mib2^cd2^, Q531stop; and Mib2^cd3^, I536stop). We selected *mib2^cd1^* and *mib2^cd3^* for further analysis because their mutation can be identified by PCR-based restriction fragment length polymorphism typing (Fig. S1). The expression of *mib2*, as examined by *in situ* hybridization in these *mib2* mutants, showed that the *mib2* mRNA level was reduced in heterozygous or homozygous mutant embryos, which was likely due to nonsense mediated decay 48 h post-fertilization (hpf) (Fig. 2A) (*mib2^cd1/+^*, 74% with low-level expression, *n*=31; *mib2^cd1^*, 85% with low-level expression, *n*=13; *mib2^cd3/+^*, 70% with low level expression, *n*=27; *mib2^cd3^*, 100%, with low-level expression, *n*=18). Both the *mib2^cd1^* and *mib2^cd3^* homozygous mutants were viable and grew to sexually mature adulthood. Therefore, the *mib2* mRNA level was further examined by quantitative real time PCR (q-PCR) using RNA extracted from *mib2^cd3^* homozygous or *mib2^cd3^* heterozygous embryos, which were obtained by crossing *mib2^cd3^* homozygous males and females or *mib2^cd3^* heterozygous males and homozygous females, respectively. This q-PCR measurement showed lower level of *mib2* expression in *mib2^cd3^* heterozygous and homozygous embryos than in wild type control embryos (Fig. 2B) (*mib2^cd3/+^*, 52%; *mib2^cd3^*, 24%). Therefore, mutations in *mib2^cd1^* and *mib2^cd3^* alleles result in a loss of *mib2* function.

### Mib2 is not involved in Notch signalling during early neurogenesis and is not redundant to Mib1

**Fig. 3.**
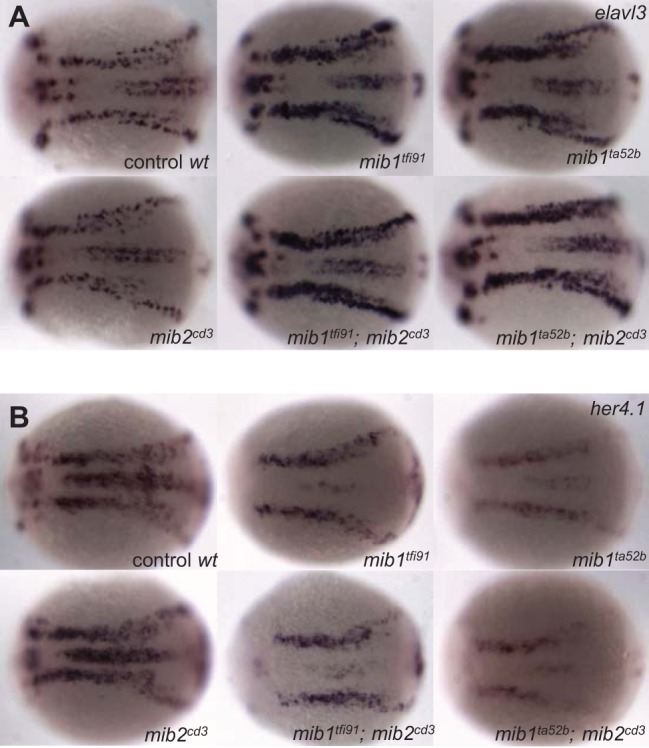
**Mib2 deficiency does not significantly affect early neurogenesis and Notch signalling.** Whole-mount *in situ* hybridization using *elavl3* (A) and *her4.1* (B) antisense probes at the 3 somite stage, showing no difference in neurogenesis phenotypes between wild-type and *mib2*^*cd3*^ embryos or between the *mib1* mutants and *mib1/mib2* double mutants. All panels show top views of embryos with the anterior to the left.

Because Mib2 exhibits sequence homology with Mib1, we investigated the functions of Mib2 alone and in collaboration with Mib1 during early neurogenesis. The expression of an early neuronal cell marker, *elavl3*, was not dramatically changed in *mib2^cd3^* mutant embryos at the 3 somite stage (Fig. 3A) (100% with normal expression, *n*=20). In contrast, embryos homozygous for two alleles of *mib1*, *mib1^tfi91^* and *mib1^ta52b^*, showed increased levels of *elavl3* expression (Fig. 3A) (*mib1^tfi91^*, 88% with high-level expression, *n*=16; *mib1^ta52b^*, 89% with high-level expression, *n*=9). We next examined the expression of a Notch target gene, *her4.1*, in the neural cells of *mib1* and *mib2* mutant embryos at the 3 somite stage (Takke et al., 1999). The expression of *her4.1* also remained unchanged in *mib2^cd3^* mutant embryos (Fig. 3B) (*mib2^cd3^*, 100% with normal expression, *n*=21), whereas it was reduced in both *mib1* mutant (*mib1^tfi91^* and *mib1^ta52b^*) embryos. However, the phenotype was slightly more pronounced in *mib1^ta52b^* embryos than in *mib1^tfi91^* embryos (Fig. 3B) (*mib1^tfi91^,* 89% with low-level expression, *n*=9; *mib1^ta52b^*, 100% with low-level expression, *n*=6). Furthermore, the collaborative functions of *mib1* and *mib*2 were examined using *mib1/mib2* double mutants. The expression levels of *elavl3* (Fig. 3A) (*mib1^tfi91^; mib2^cd3^*, 92% with high-level expression, *n*=12: *mib1^ta52b^; mib2^cd3^*, 75% with high-level expression, *n*=8) and *her4.1* (Fig. 3B) (*mib1^tfi91^; mib2^cd3^*, 83% with low-level expression, *n*=12: *mib1^ta52b^; mib2^cd3^*, 100% with low-level expression, *n*=7) did not significantly differ between the *mib1* mutants and *mib1/mib2* double mutants. These data suggest that Mib2 does not play a significant role in Notch signalling during early neurogenesis, alone or in combination with Mib1.

### Mib2 does not exert maternal functions during early neurogenesis and somitogenesis and is not redundant to Mib1

**Fig. 4.**
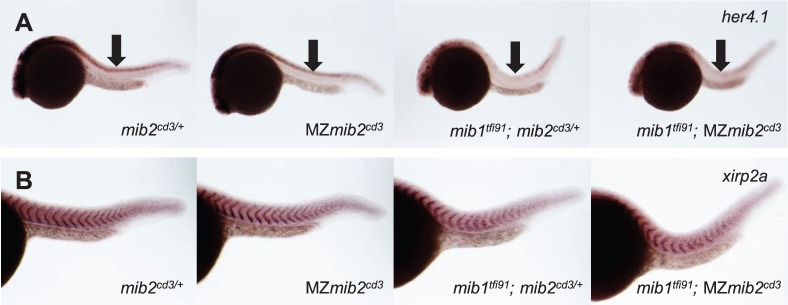
**Maternal Mib2 does not compensate for the zygotic loss of Mib2.** Whole-mount *in situ* hybridization using *her4.1* (A) and *xirp2a* (B) antisense probe at 24 hpf. Maternal deletion of *mib2* does not affect expression of *her4.1* or *xipr2a*. Whole embryo (A) and trunk region (B) are shown. Arrows show expression of *her4* in the trunk neural tube. Side views of embryos with anterior to the left.

Previous studies have shown that the maternal expression of genes can partially compensate for a loss of zygotic gene function (Gritsman et al., 1999; Mintzer et al., 2001). Because *mib2* is maternally expressed, we addressed the maternal function of Mib2. Homozygous *mib2^cd3^* embryos survived to fertile adulthood, and maternal-zygotic (MZ) *mib2^cd3^* mutant embryos were obtained by mating homozygous *mib2^cd3^* females with heterozygous *mib2^cd3^* males. In MZ *mib2^cd3^* mutant embryos, *her4.1* expression at 24 hpf did not differ from that in heterozygous control embryos (Fig. 4A) (MZ *mib2^cd3,^* 100% with normal expression, *n*=11). We also investigated the maternal function of Mib2 during early somitogeneis, but the pattern of somite segmentation, as revealed by *xirp2a* expression, was not affected in MZ *mib2^cd3^* mutant embryos at 24 hpf (Fig. 4B) (number of somite boundaries; *mib2^cd3/+^*, 19.1±0.5, *n*=11; MZ *mib2^cd3^*, 19.3±1.4, *n*=11). In contrast, *her4.1* expression was reduced in *mib1^tfi91^*; *mib2^cd3/+^* embryos (Fig. 4A) (*mib1^tfi91^;* *mib2^cd3/+^*, 100% with low-level expression, *n*=10), and the regular spacing of the somites was disrupted (Fig. 4B) (number of boundaries: *mib1^tfi91^;* *mib2^cd3/+^*, 16.4±2.3, *n*=16) because these deficits are due to the disruption of Notch function. However, *mib1^tfi91^;* MZ *mib2^cd3^* double mutant embryos did not show more severe phenotypes than *mib1^tfi91^; mib2^cd3/+^* embryos, both in *her4.1* expression (Fig. 4A) (*her4.1*: *mib1^tfi91^;* MZ*mib2^cd3^*, 100% with a similar level to that in *mib1^tfi91^*; *mib2^cd3/+^*, *n*=5) and somite numbers (Fig. 4B) (*mib1^tfi91^;* MZ*mib2^cd3^*, 16.1±1.1, *n*=14). Therefore, maternal *mib2* expression does not compensate for the loss of zygotic Mib2 function.

### Mib2 is not involved in melanophore development and does not antagonize Mib1^ta52b^ protein

**Fig. 5.**
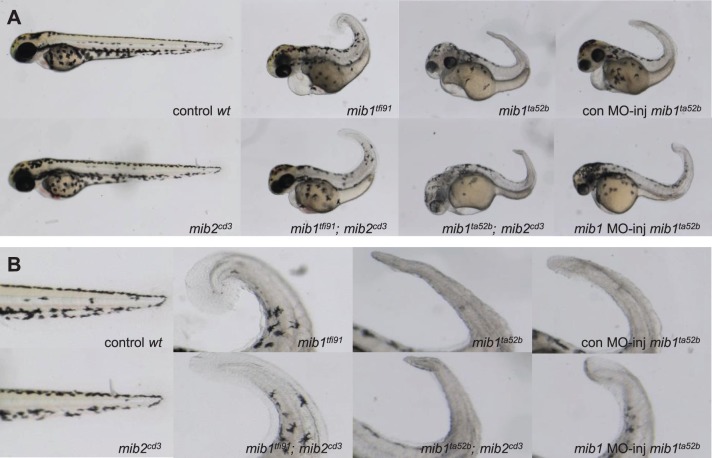
**Mib2 neither regulates melanophore development nor antagonizes Mib1^ta52b^ protein.** Whole embryos (A) or enlarged views (B) of tail region in A. *mib2* deletion did not enhance pigmentation loss in *mib1^tfi91^*, nor did it recover pigmentation in *mib1^ta52b^* mutants. All panels show side views of embryos with anterior to the left.

A loss of Mib1 function results in a white tail phenotype caused by a decrease in neural crest-derived black melanophores. As reported earlier, we observed the white tail phenotype in the *mib1* mutants 2 days post fertilization (dpf), and the phenotype was more severe in *mib1^ta52b^* than in *mib1^tfi91^* embryos (Fig. 5A,B) (*mib1^tfi91^*, 100% with mild phenotype, *n*=7; *mib1^ta52b^*, 100% with severe phenotype, *n*=10). In contrast, the tail pigmentation was normal in *mib2^cd3^* mutant embryos, and the pigment phenotype was not enhanced in *mib1^tfi91^; mib2^cd3^* double mutant embryos (Fig. 5A,B) (*mib2^cd3^*, 100% with normal phenotype, *n*=18: *mib1^tfi91^; mib2^cd3^,* 100% with similar phenotype to *mib1^tfi91^*, *n*=11).

Zhang et al. reported the dominant-negative effects of Mib1^ta52b^ (M1013R) protein on Mib1 and Mib2 proteins. Specifically, they observed that a *mib1*-MO injection rescues *mib1^ta52b^* mutants to a *mib1^tfi91^*-like phenotype, and *mib2*-MO-injected *mib1^tfi91^* mutants phenocopy *mib1^ta52b^* mutants. We observed a reduction of pigmentation in *mib1^ta52b^* mutants, which was rescued by *mib1*-MO injection (Fig. 5A,B) (control-MO injected *mib1^ta52b^* vs *mib1*-MO injected *mib1^ta52b^*, 100% rescued, *n*=7 and *n*=5, respectively). However, a loss of Mib2 did not recover pigmentation in *mib1^ta52b^* mutants (Fig. 5A,B) (*mib1^ta52b^; mib2^cd3^,* 100% not rescued, *n*=13). These results suggest that Mib2 does not play important roles in Notch signalling, which regulates pigment cell development. Furthermore, Mib2 does not influence the antimorphic effects of Mib1^ta52b^ protein.

### Notch-independent roles of Mib2 in muscle and NMDAR activity are not apparent *in vivo*

**Fig. 6.**
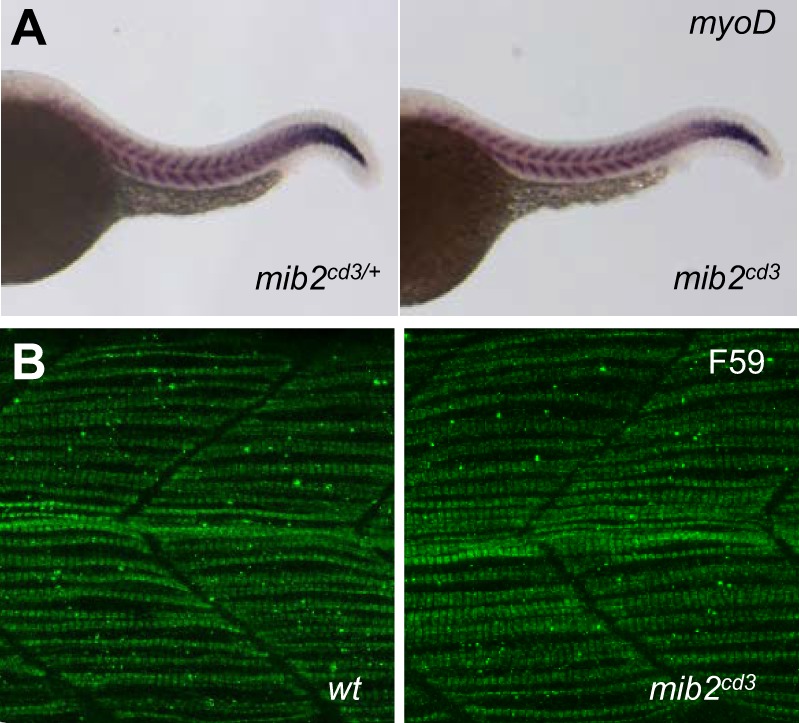
**Muscle structure is normal in *mib2* mutant embryos.** (A) Whole-mount *in situ* hybridization using the *myoD* in trunk region. (B) Slow muscle fibre myosin in trunk muscles as revealed by F59 antibody. *mib2* mutant embryos did not show any changes in the pattern or level of their staining. All panels show side views of embryos with anterior to the left at 24 hpf.

Mib2 is assumed to be involved in muscle integrity in zebrafish, as reported for *Drosophila* (Nguyen et al., 2007; Carrasco-Rando and Ruiz-Gomez, 2008). Therefore, we examined slow and fast muscle formation by detecting the levels of *myoD* mRNA and myosin heavy chain protein at 24 hpf. The level of *myoD* mRNA, which is expressed in both slow and fast muscles, was not dramatically changed in MZ *mib2^cd3^* mutant embryos (Fig. 6A) (100% with normal expression, *n*=18). Likewise, slow muscle formation was not dramatically affected in *mib2^cd3^* mutants, as detected by F59 antibody, which recognizes myosin heavy chain protein (Fig. 6B) (100% with normal expression, *n*=5).

**Fig. 7.**
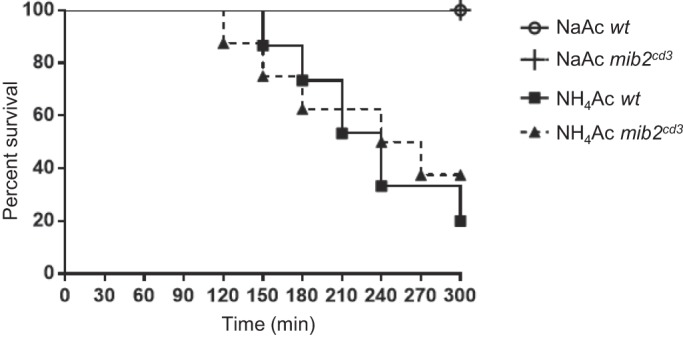
**Mib2 deficiency does not affect survival of 4 dpf embryos exposed NH_4_Ac.** Embryos were monitored for their heartbeats every 30 min. NaAc, sodium acetate-treated groups. NH_4_Ac, ammonium acetate-treated groups. The survival of *mib2^cd3^* embryos was comparable to that of wild type control embryos by NH_4_Ac treatment. NaAC wild type, *n*=14; NH_4_Ac wild type, *n*=15. NaAC *mib2^cd3^*, *n*=7; NH_4_Ac *mib2^cd3^*, *n*=8.

Previously, Jurd et al. suggested that Mib2 negatively regulates the functional activity of NMDAR by ubiquitinating its NR2B subunit (Jurd et al., 2008). Unfortunately, we could not measure the NR2B protein level in the *mib2^cd3^* mutant due to the unavailability of zebrafish NR2B-specific antibody. Therefore, we utilized an ammonia toxicity assay to evaluate NMDAR activity in zebrafish. Hyperammonaemia leads to elevated extracellular glutamate concentrations, which hyperstimulates NMDAR (Kosenko et al., 2003). Several NMDAR antagonists prevent hyperammonaemia-induced death in zebrafish (Feldman et al., 2014). As reported previously, we observed that ammonium acetate (NH_4_Ac) treatment significantly decreased survival compared with the control sodium acetate (NaAc) treatment (Fig. 7) (NaAC WT vs NH_4_Ac WT; *P*<0.001). However, NH_4_Ac treatment did not reduce the survival of *mib2^cd3^* embryos compared with that of wild type control embryos (Fig. 7) (NH_4_Ac *wt* vs NH_4_Ac *mib2^cd3^*; *P*=0.61).

These results suggested that Mib2 might not regulate muscle integrity and NMDAR activity in zebrafish during embryogenesis.

## DISCUSSION

### Mib2 does not play important roles in Notch signalling during development

Previous studies show that Mib2 ubiquitinates the Delta and Jagged proteins to enhance their endocytosis *in vitro* (Koo et al., 2005; Takeuchi et al., 2005). These actions are similar to that of Mib1, a paralogue of Mib2, which is known to be essential for activating Notch signalling *in vivo*. Here, we show that Mib2 is dispensable, at least during neurogenesis, somitogenesis, and pigment cell development, and for Notch signalling in zebrafish. Similar observations have been made in the mouse and *Drosophila* (Koo et al., 2007; Nguyen et al., 2007; Wu et al., 2007). Furthermore, our data suggest that the maternal gene product of Mib2 does not play a critical role during zebrafish development. These studies suggest that Mib2 is not essential for activating Notch signalling via the ubiquitination of Notch ligands during metazoan development, although we cannot exclude the possibility that Mib2 functions at later stages or in tissues that were not examined in this study.

### Redundancy between Mib1 and Mib2 functions

Because Mib1 and Mib2 share substrates and form hetero-oligomers *in vitro*, they may act in a redundant fashion (Koo et al., 2005; Takeuchi et al., 2005; Zhang et al., 2007a). However, functional redundancy between Mib1 and Mib2 *in vivo* has been controversial in different species. Koo et al. reported that Mib1 and Mib2 do not act redundantly to control mouse embryonic development because a Mib1/Mib2 double knockout does not enhance the phenotypes of Mib1 knockout mice (Koo et al., 2007). In contrast, Zhang et al. suggested that these proteins play redundant roles based on the characterization of zebrafish antimorphic *mib1* alleles (Zhang et al., 2007b).

In zebrafish, a phenotypic comparison of different *mib1* alleles revealed that *mib^ta52b^* (Mib1-M1013R) results in more severe defects than those of other alleles that produce a premature stop codon, such as *mib^tfi91^* (Zhang et al., 2007b). Therefore, *mib^ta52b^* is an antimorphic but not dominant-negative allele because it is inherited in a recessive manner. This recessive antimorphism is a rare genetic phenomenon and was recently reported for the strongest allele of the TSO1 gene in *Arabidopsis* (Sijacic et al., 2011). The recessive antimorphic allele can produce a phenotype more severe than null by interfering with the function of family genes. In accordance with this function, Zhang et al. suggested that Mib2 may be involved in the more pronounced phenotype in *mib1^ta52b^*, i.e. the roles of Mib2 and Mib1 are redundant, because *mib1*-null mutants (*mib^tfi91^*) with *mib2* knockdown phenocopy *mib^ta52b^* mutants and *mib2* mRNA injection partially rescues *mib^ta52b^* mutant phenotypes. On the contrary, our study showed that the genetic ablation of *mib2* neither enhances *mib^tfi91^* phenotypes nor alleviates *mib^ta52b^* phenotypes, suggesting that the actions of endogenous Mib2 and Mib1 are not redundant.

Two possibilities may account for this discrepancy. First, the truncated Mib2 protein, which is produced by the *mib2^cd3^* allele but not by the *mib2*-MO knockdown, might exert residual functions to support Mib1/Mib2 hetero-oligomer activity. However, this possibility is unlikely because the level truncated *mib2^cd3^* protein itself may be reduced due to its mRNA decay. Second, other protein(s) may compensate for a loss of Mib2 function in the *mib2^cd3^* allele during development, but this developmental compensation is precluded by morpholino-mediated *mib2* knockdown. Supporting the this notion, Rossi et al. recently reported the activation of a compensatory network to buffer against genetic deleterious mutations, which was not observed after translational or transcriptional knockdown (Rossi et al., 2015). Future studies should investigate the compensatory activation of genes in *mib2^cd3^* mutants.

### Notch signalling-independent functions of Mib2

Several Notch-independent functions of Mib2 have been reported (Nguyen et al., 2007; Jurd et al., 2008; Stempin et al., 2011). In *Drosophila*, Mib2 plays a Notch-independent role in muscle integrity and survival (Nguyen et al., 2007; Carrasco-Rando and Ruiz-Gomez, 2008). However, our study and others show that in zebrafish, Mib1 but not Mib2 is sufficient for the maintenance of somite integrity (Pascoal et al., 2013). Therefore, the role of Mib family proteins in the muscular system may differ by species.

The NR2B subunit of the NMDAR is a potential substrate for Mib2 and is negatively regulated by Mib2 in a ubiquitin-proteasome-dependent manner *in vitro* (Jurd et al., 2008). In the absence of Mib2 function, NMDAR activity might be upregulated due to the increased NR2B protein level. However, Mib2 deficiency does not affect NMDAR activity in zebrafish embryos, as assessed based on the neurotoxic effects of ammonia, suggesting that Mib2 may not be involved in NMDAR activity. However, one caveat associated with this interpretation is that the ammonia toxicity assay is not sufficiently sensitive to determine the effect of Mib2 on NMDAR activity *in vivo*. Future studies should address this issue.

## MATERIAL AND METHOD

### Zebrafish lines and maintenance

The zebrafish were raised and maintained under standard conditions with approval by the Institutional Animal Care and Use Committee at Chiba University. Zebrafish embryos were obtained from the natural spawning of wild-type adults or identified carriers, which were heterozygous for *mib2^cd1^*, *mib2^cd3^*, *mib1^tfi91^; mib2^cd3^*, *mib1^ta52b^; mib2^cd3^*, and *mib1^tfi91^* and homozygous for *mib2^cd3^*.

### Construction of TALEN plasmids

The plasmids used to synthesize TALEN mRNAs were constructed as described previously (Hisano et al., 2015). RVD repeat arrays were cloned into pCS2TAL3DD and pCS2TAL3RR to generate left and right TALEN constructs (*mib2*-TALEN-F and *mib2*-TALEN-R). The amino acid sequences of the constructed TALENs are shown in Table S1.

### mRNA and morpholino antisense oligonucleotide injection

To microinject TALEN mRNA, the *mib2*-TALEN-F and *mib2*-TALEN-R plasmid were linearized and transcribed with SP6 RNA polymerase using the mMessage mMachine Kit (Life Technologies). The *mib2* forward and reverse TALEN mRNAs (400 pg each) were injected together into the blastomeres of zebrafish embryos at the one-cell stage. The Mib1 morpholino was obtained from Gene Tools and used as described previously (Itoh et al., 2003).

### Whole-mount *in situ* hybridization and antibody staining

Whole-mount *in situ* hybridization was performed as described previously (Yamamoto et al., 2010). The zebrafish *mib2* probe was generated from a pCR TOPOII vector plasmid in which the *mib2* cDNA fragment was subcloned. All probes have been previously published: *her4.1* (Takke et al., 1999), *elavl3* (Kim et al., 1996)*, xirp2a* (Schröter and Oates, 2010). Whole-mount antibody staining was performed using the following antibodies: anti-Myosin heavy chain (F59, DSHB) and Alexa-488 anti-mouse IgG (Invitrogen).

### PCR-based restriction fragment length polymorphism typing

Genotyping was performed using following primers and restriction enzymes. Fragments were confirmed by electrophoresis.

*cd1*: Fw, CTGCTACAGGCTAACAGTAATAT and Rev, ATAACGATTTCTGCAGCGAAG. Restriction enzyme, *Ssp*I (TAKARA BIO, Japan). Fragment size: wild type, 130 bp; heterozygote, 150+130 bp; homozygote, 150 bp. *cd3*: Fw, GGTGGACATTAAGAACCAGGGAAAG and Rev, GCGCCGCGTCTCCGTCCTCATCTTAAGCT. Restriction enzyme, *Hin*dIII (TOYOBO, Japan). Fragment size: wild type, 100 bp; heterozygote, 130 bp+100 bp; homozygote; 130 bp.

### Quantitative RT–PCR (q-PCR)

Total RNA was obtained using RNAiso Plus (TaKaRa Bio, Japan) according to the manufacturer's protocol. Total RNA was reverse transcribed using ReverTra Ace (TOYOBO) according to the manufacturer's protocol. The zebrafish *mib2* gene sequence was retrieved from the UCSC Genome Browser for real time PCR (http://genome.ucsc.edu/cgi-bin/hgGateway). The primers were designed by Primer3web version 4.0.0 (http://primer3.ut.ee/). Primer specificity was confirmed by NCBI/Primer-BLAST (http://www.ncbi.nlm.nih.gov/tools/primer-blast/). The transcript levels of *mib2* and *Rpl13α* were quantified by real-time PCR with Power SYBR Mix (Applied Biosystems) on a 7300 Real-Time PCR detection system (Applied Biosystems) as described previously (Itoh et al., 2014).

### Ammonia toxicity assay

One dpf embryos were arrayed in a 12-well plate with 3 ml of E3 medium (5 mM NaCl, 0.17 mM KCl, 0.33 mM CaCl_2_, and 0.33 mM MgSO_4_). At 4 dpf, the larvae were treated with sodium acetate (NaAc; Wako, Japan) or ammonium acetate (NH_4_Ac; Wako). The survival time was assessed every 30 min based on the presence/absence of a visible heartbeat. The Mantel–Cox log-rank test was used to statistically analyse the data using the Prism 6 software (GraphPad).
